# Exploring the Utilization of PHC Pile Waste Concrete as Filler in Asphalt Mastics

**DOI:** 10.3390/ma16227158

**Published:** 2023-11-14

**Authors:** Botao Tu, Xinkui Yang, Shi Xu, Xuhui Liang, Chen Liu, Jian Jiang, Lulu Fan, Liangliang Tu

**Affiliations:** 1 Guangdong Hongye Building Materials Technology Co., Ltd., Yunfu 527121, China; botao7659@163.com; 2 State Key Laboratory of Silicate Materials for Architectures, Wuhan University of Technology, Wuhan 430070, China; 3 School of Civil Engineering and Architecture, Wuhan University of Technology, Luoshi Road 122, Wuhan 430070, China; xushi@whut.edu.cn; 4 Faculty of Civil Engineering and Geosciences, Delft University of Technology, Stevinweg 1, 2628 CN Delft, The Netherlands; x.liang-1@tudelft.nl (X.L.); c.liu-12@tudelft.nl (C.L.); 5 Shenzhen Sez Construction Group Co., Ltd., Shenzhen 518034, China; jiangjian@szcg.cn (J.J.); fanlulu410@163.com (L.F.); tlliang@whut.edu.cn (L.T.)

**Keywords:** PHC pile waste concrete, filler, particle characteristics, asphalt mastics, physical properties, rheological properties, low-temperature crack resistance

## Abstract

Using solid waste to replace limestone filler in asphalt concrete can not only reduce the cost of road construction, but also improve the utilization rate of solid waste. In this study, PHC pile waste concrete (PPWC) was innovatively used to replace limestone filler in asphalt mixture and its effect on the physical and rheological properties of asphalt mastics was studied. Firstly, PPWC was ground into filler particles with a diameter less than 0.075 mm. The physical properties, particle characteristics and chemical composition of PPWC filler and limestone filler were compared. Asphalt mastics were prepared with different filler-asphalt volume ratios (20%, 30% and 40%) and the physical properties, high-temperature rheological properties and low-temperature cracking resistance of asphalt mastics were tested. The experimental results showed that the surface of PPWC filler is rougher and has lower density and smaller particle size than limestone filler. When the filler content is the same, PPWC filler asphalt mastics have lower penetration and ductility, higher softening point than limestone filler asphalt mastics, and the viscosity of PPWC filler asphalt mastics is more sensitive than limestone filler asphalt mastics. PPWC filler asphalt mastics demonstrated superior high-temperature stability, but poorer low-temperature cracking resistance compared to limestone filler asphalt mastics. In conclusion, PPWC fillers can be used to replace limestone fillers in asphalt mixtures. The finding of this study will provide a new solution for the construction of eco-friendly roads.

## 1. Introduction

As a kind of hollow cylindrical precast concrete component, prestressed high-strength concrete pile (PHC pile) is widely employed in the foundations of various construction, including highways and railroads [1,2,3]. China is the country with the highest number of PHC pile manufacturers in the world. In 2022, China’s PHC pile production reached 486.28 million meters [4]. However, PHC pile is typically installed underground, and after long-term erosion caused by groundwater, it is susceptible to structural damage, leading to a loss of load-bearing capacity [5]. The accumulation of discarded PHC pile leads to the waste of substantial land resources. Sua-iam and Makul [6] prepared an environmentally friendly self-compacting concrete using PHC pile waste concrete (PPWC) as aggregate and FA as cementitious material. They found that PPWC aggregates reduce the viscosity, fluidity and compressive strength of the concrete, but the corrosion risk of concrete is enhanced. However, there are still few studies on the reuse of PPWC. Therefore, the utilization of PPWC has become an urgent issue that needs to be addressed.

As the primary material of pavement structure at present, asphalt concrete has the advantages of high load-bearing capacity, excellent skid resistance, and effective noise reduction [7,8,9,10,11]. As a result, it is extensively employed in high-quality pavement around the world [12,13,14,15]. Asphalt concrete is produced by compacting asphalt mixtures, which can be divided into two main components: asphalt mastics and aggregates [16,17,18,19]. The asphalt mastics are the combination of mineral filler and asphalt binder [20,21]. The asphalt mastics are uniformly wrapped on the surface of aggregate and filled in the gap between aggregate. When the asphalt concrete is subjected to external forces, the asphalt mastics will first exhibit strain behavior. Therefore, the macroscopic and viscoelastic characteristics of asphalt concrete are greatly affected by asphalt mastics [22,23]. The mineral filler can strengthen the uniformity of the asphalt and fill the voids in the asphalt concrete, increasing its strength [24,25,26]. Limestone filler is the most commonly used mineral filler [27]. However, mineral resources are continuously consumed as a result of the road industry’s rapid development, leading to increased costs and environmental degradation associated with limestone extraction [28,29,30]. Therefore, how to reduce the cost of engineering construction while minimizing reliance on natural resources has become a pressing concern for the road industry [31].

Some low-utilization geotechnical materials have been utilized to prepare asphalt concrete as filler [32,33,34]. The rheological characteristics of asphalt mastics were studied by Zhao et al. [35] using calcareous sand to replace limestone fillers in asphalt concrete. They found that the fundamental characteristics of calcareous sand filler are similar to limestone filler, as well as improving asphalt mastics’ low temperature performance and resilience to fatigue. Islam et al. [36] used waste jarosite as the substitute for limestone filler in asphalt concrete and evaluated how waste jarosite filler affected the asphalt mixture’s engineering characteristics. It was discovered that adding waste jarosite filler to the asphalt concrete increased its indirect tensile strength and resistance to rutting, while the fatigue life of the asphalt concrete was decreased. Boateng et al. [37] used montmorillonite as filler to replace different contents of limestone filler in asphalt concrete, and studied the pore characteristics and stability of asphalt concrete. They found that montmorillonite filler can greatly increase asphalt concrete’s density and stability, which may be due to the higher friction between the particles of the filler. But montmorillonite filler will weaken the material’s resistance to rutting in high temperatures. Furthermore, numerous studies have also reported the substitution of solid waste for mineral filler in asphalt mixtures [38,39,40], including industrial solid waste, metallurgical solid waste, agricultural solid waste and construction solid waste [41,42,43]. Li et al. [44] pretreated phosphogypsum at different temperatures and used it as the replacement for limestone filler in asphalt mixture. They found that although decreasing the fatigue life of asphalt mastics, the performance of asphalt mastics at high temperatures can be improved with phosphogypsum filler. Zhang et al. [45] substituted steel slag powder (SSP) for the limestone filler in asphalt mastics and studied the creep and fatigue properties of asphalt mastics. They found that SSP had high alkalinity, furthermore, the incorporation of SSP can effectively enhance the asphalt mastics’ high temperature stability and fatigue resistance. Rice husk ash (RHA) and date seed ash (DSA) were employed by Tahami et al. [46] as an alternate filler for hot mix asphalt mixture, additionally, the asphalt mixture’s performance on roads was evaluated. The results demonstrated that RHA and DSA can increase the asphalt mixture’s fatigue resistance while simultaneously improving its Marshall stability and rutting resistance. Lei et al. [47] used recycled concrete powder (RCP) as filler in asphalt concrete, and studied how RCP content affected the performance of asphalt mastics. The results show that as RCP concentration increased, asphalt mastics’ softening point increased while its penetration and ductility reduced. Compared with limestone filler, the resistance of the asphalt mastics to rutting at high temperatures can be improved by using RCP filler.

In summary, the use of solid waste materials in place of mineral fillers can increase the utilization of waste resources, lower the cost of raw materials, and improve the working properties of asphalt concrete. However, the research on using PPWC as the filler in asphalt mixtures remains limited, and it is still unclear how PPWC filler may affect the rheological performance and physical characteristics of asphalt mastics.

This study used PPWC as filler to prepare asphalt mastics to evaluate the feasibility of replacing the limestone filler in asphalt mixture with PPWC filler. Firstly, by using the planetary ball mill, PPWC was crushed into powder with a particle size less than 0.075 mm. Both the PPWC filler and the limestone filler’s chemical composition and particle characteristics were investigated. Then, different volume fractions of limestone filler and PPWC filler were uniformly mixed with matrix asphalt by high-speed shearing apparatus to obtain asphalt mastics. The softening point, penetration, ductility and viscosity of asphalt mastics were tested. Furthermore, using bending beam rheometers (BBR) and dynamic shear rheometers (DSR), the rheological characteristics of asphalt mastics at low and high temperatures were investigated. The study findings will offer a theoretical foundation for using PPWC filler in asphalt concrete.

## 2. Raw Materials and Preparation of Asphalt Mastics

### 2.1. Raw Materials

**Figure 1:**
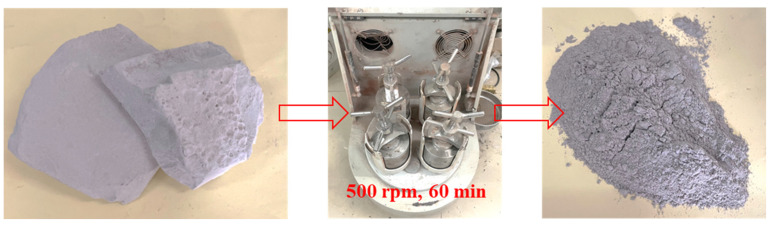
The preparation process of PPWC filler.

The base asphalt for this study was 70# asphalt produced in Hebei Province, China. The results of the evaluation of its basic properties in line with the Chinese standard JTG E20-2011 [48] are presented in Table 1. Limestone filler was sourced from the quarry in Wuhan, Hubei Province, China, while the PPWC was obtained from a PHC pile manufacturer in Yunfu, Guangdong Province, China. PPWC was ground into particles with a diameter smaller than 0.075 μm at a speed of 500 rpm, as shown in Figure 1.

**Figure 2:**
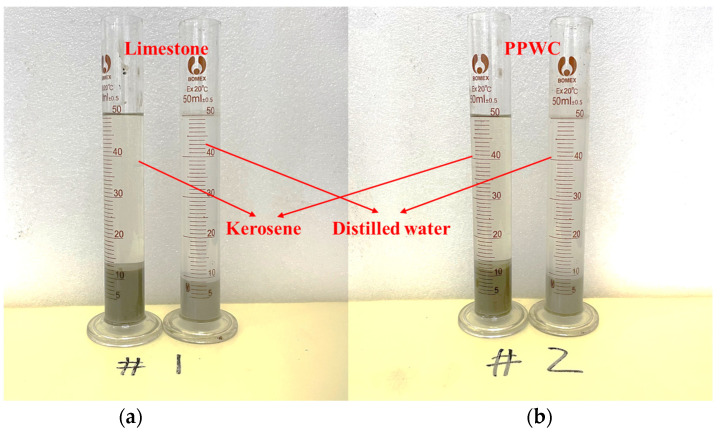
Hydrophilicity coefficient test, (**a**) Limestone, (**b**) PPWC.

The basic properties of PPWC and limestone filler were assessed in accordance with Chinese standard JTG-E42-2005 [49], and the results are presented in Table 2. It is evident that the PPWC filler has a slightly lower density than the limestone filler. The volume of the filler in water to its volume in kerosene is the hydrophilic coefficient, which is used to assess the adhesive qualities of the filler to asphalt binder. The testing process for the hydrophilic coefficient is illustrated in Figure 2. The results show that the hydrophilic coefficient of PPWC is 0.79, indicating that the affinity of PPWC to asphalt is higher than that to water, which meets the requirements of the standard.

### 2.2. Preparation of Asphalt Mastics

**Figure 3:**
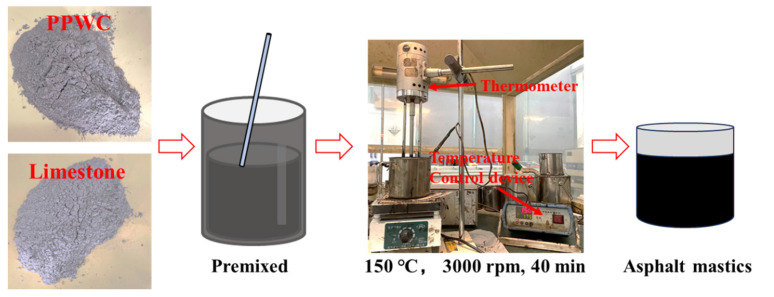
Preparation process of asphalt mastics.

Firstly, the base asphalt was heated at 160 °C for 40 min, and the two fillers were heated at 120 °C for 60 min to remove excess water present in the fillers. Then, the two fillers were slowly added to the asphalt at 20%, 30% and 40% of the asphalt volume, respectively, and pre-stirred with a glass rod for 10 min. After the filler is initially dispersed, the high-speed shear apparatus was used to shear the asphalt mastics at 150 °C for 40 min at 3000 rpm to guarantee that the filler in the asphalt mastics was evenly and completely dispersed. Figure 3 depicts the method of preparing asphalt mastics.

## 3. Test Methods

### 3.1. Particle Characteristics and Chemical Composition of Fillers

The particle characteristics of limestone filler and PPWC filler were analyzed by scanning electron microscopy (SEM, Zeiss Gemini 300, Carl Zeiss AG, Jena, Germany) and laser particle size analyzer (Mastersizer 3000, Malvern Panalytical, Herrenberg, Germany). The chemical composition and phase distribution of the two fillers were analyzed by X-ray fluorescence (XRF) test and X-ray diffraction (XRD) test. According to the study of Gautam et al. [50], a layer of gold was plated on the surface of the sample before the SEM test to improve its conductivity. During the XRD test, the sample must be crushed into powder with a particle size of less than 90 μm.

### 3.2. Physical Properties of Asphalt Mastics

The penetration, softening point, and ductility of asphalt mastics were evaluated in accordance with Chinese standard JTG E20-2011 to evaluate the impact of two fillers on the physical characteristics of asphalt mastics, at 25 °C for the penetration test and 5 °C for the ductility test, respectively. The Brookfield viscosity of asphalt mastics was also tested at five temperatures (105 °C, 120 °C, 135 °C, 150 °C and 165 °C) to evaluate the machinability of asphalt mastics.

### 3.3. Rheological Properties of Asphalt Mastics

Asphalt mastics were subjected to a temperature sweep test by DSR. The high-temperature rheological properties of asphalt mastics were analyzed by complex modulus (G*), phase angle (δ) and rutting factor (G*/sinδ). The temperature range was set to 30 °C–80 °C, the heating rate was set to 2 °C/min, and the frequency was set to 10 rad/s.

The frequency range of 0.01–10 Hz was used for the frequency sweep test of asphalt mastics, and the viscoelastic behavior of asphalt mastics was analyzed by G*. All tests were carried out at five temperatures of 46 °C, 52 °C, 58 °C, 64 °C, and 70 °C.

### 3.4. Low-Temperature Crack Resistance of Mastics

The low-temperature crack resistance of asphalt mastics was studied by BBR test. The test temperature was set to −12 °C, −18 °C, and −24 °C. Continuous load was applied to the beam specimen prepared by asphalt mastics at low temperature, and the creep stiffness (S) and m-value (m) were obtained by the creep response of the beam specimen.

## 4. Results and Discussions

### 4.1. Characteristics of Filler

#### 4.1.1. Surface Morphology of Filler

**Figure 4:**
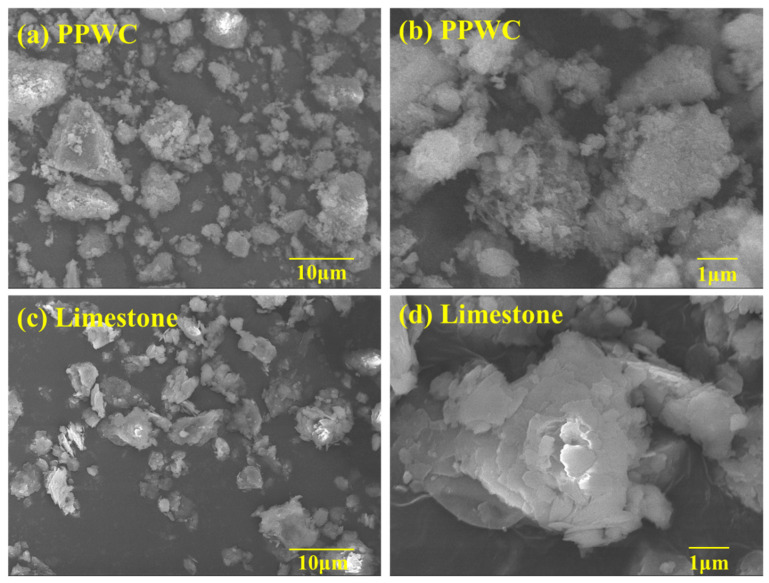
SEM images of PPWC filler and limestone filler, (**a**) PPWC filler magnified by 2000 times, (**b**) PPWC filler magnified by 10,000 times, (**c**) Limestone filler magnified by 2000 times, (**d**) Limestone filler magnified by 10,000 times.

The SEM images of PPWC filler and limestone filler are shown in Figure 4. Figure 4a,b show the micro-morphology of PPWC filler after magnification of 2000 times and 10,000 times, respectively. Figure 4c,d show the micro-morphology of limestone filler after magnification of 2000 times and 10,000 times, respectively. It can be observed that the PPWC filler consists of irregularly shaped particles, and compared to the limestone filler, the PPWC filler has smaller particle sizes and a rougher surface. In contrast, the surface of the limestone filler is composed of smooth irregular flakes. According to the study of Xing et al. [51]. The rougher the surface of the filler, the stronger the interlocking effect between the filler and the asphalt. Therefore, filler with rougher surfaces is advantageous in improving the density, strength and skid resistance of asphalt concrete.

#### 4.1.2. Particle Size Distribution of Filler

**Figure 5:**
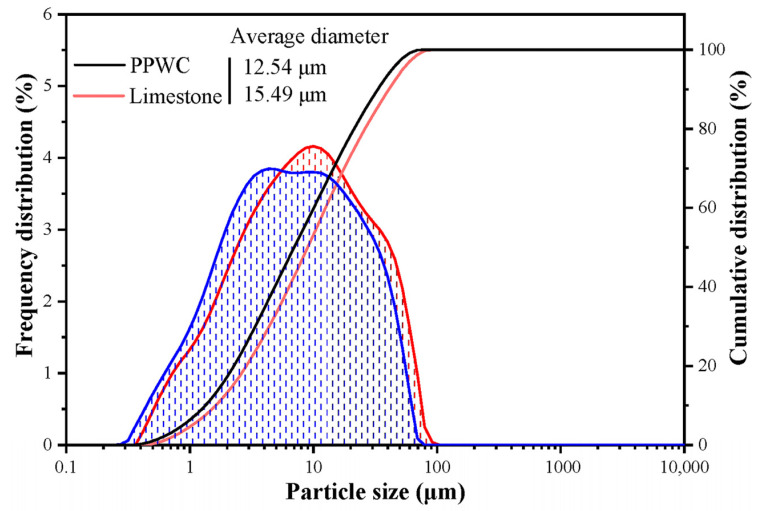
Particle size distribution curves of the two fillers.

The particle size distribution curves of the two fillers are shown in Figure 5. The results show that the average particle size of PPWC filler is 12.54 μm, which is smaller than that of limestone filler (15.49 μm). Table 3 shows the specific surface area of the two fillers and the diameters corresponding to the 10%, 50%, and 90% on the cumulative distribution curve. The results show that the specific surface area of PPWC filler (1.77 m^2^/g) is higher than that of limestone filler (1.44 m^2^/g). This may be because PPWC filler not only has smaller average particle size and density than limestone filler, but also has rougher surface than limestone filler.

#### 4.1.3. Chemical Composition of Filler

Table 4 displays the result of the XRF tests conducted on the PPWC and limestone fillers. It is evident that CaO and SiO_2_ are the primary oxide in both PPWC filler and limestone filler. Limestone filler is primarily composed of calcium carbonate, resulting in a higher CaO content (56.92%) than that of PPWC (35.10%). In contrast, PPWC filler exhibits a higher SiO_2_ content (32.4%) compared to limestone filler (18.35%), which may be because PPWC contains aggregates rich in SiO_2_.

**Figure 6:**
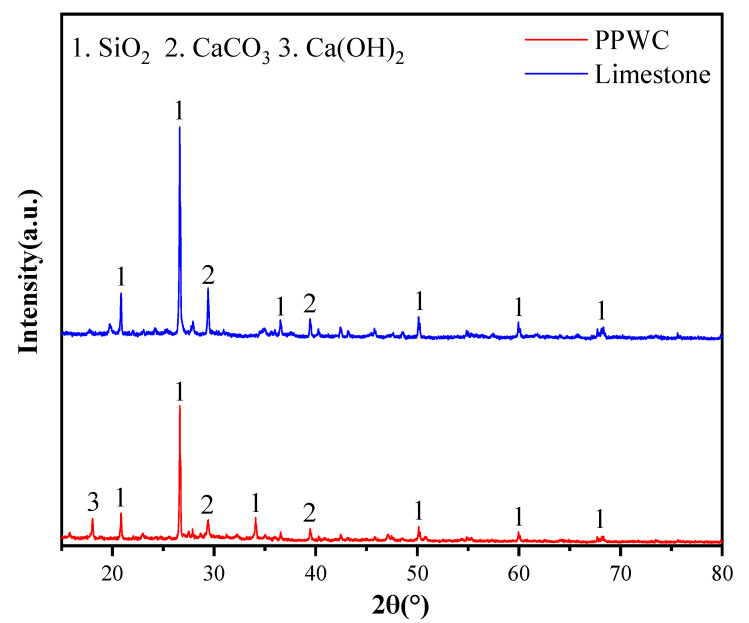
XRD curves of PPWC filler and limestone filler.

The XRD curve of PPWC filler and limestone filler are presented in Figure 6. The results show that the two fillers’ mineral composition are similar, with the primary crystalline phases being SiO_2_ and CaCO_3_. However, the XRD curve of PPWC also shows the diffraction peak of Ca(OH)_2_. According to the study of Han et al. [52], this may be attributable to the cementitious materials’ hydration during PHC pile production.

### 4.2. Physical Properties of Asphalt Mastics

#### 4.2.1. Softening Point, Penetration and Ductility of Asphalt Mastics

**Figure 7:**
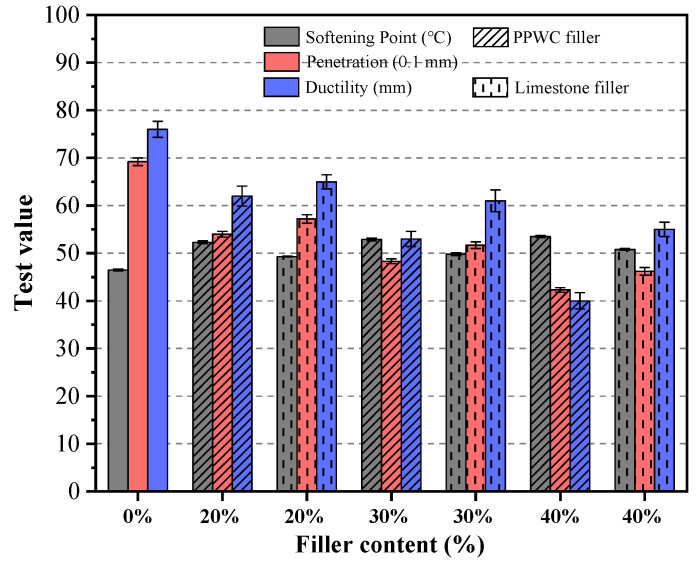
Softening point, penetration and ductility test results of asphalt mastics.

The softening point, penetration, and ductility test results of asphalt mastics are shown in Figure 7. The penetration and ductility of the asphalt mastics decreased as the filler content increased, although the asphalt mastics’ softening point increased. As the filler-to-asphalt volume ratio increased from 0% to 40%, the softening point of the asphalt mastics containing PPWC filler improved by 15.1%, but the penetration and ductility decreased by 38.9% and 47.4%, respectively. Additionally, the penetration and ductility of the asphalt mastics containing limestone filler reduced by 33.2% and 27.6%, respectively, while the softening point increased by 9.2%. Despite it having the same filler content, asphalt mastics with PPWC filler exhibited a higher softening point than those with limestone filler, and the penetration and ductility are lower than that of the limestone filler, indicating that PPWC filler can more effectively enhance the rigidity of the base asphalt compared to limestone filler. The study results of Chen et al. [53] show that increasing the rigidity of asphalt mortar can significantly improve the strength of asphalt concrete.

#### 4.2.2. Viscosity of Asphalt Mastics

The Brookfield viscosity test, as one of the characterization methods for asphalt performance, can assess the processability of asphalt mastics and reflect the high-temperature stability of asphalt mastics. According to the research of Gong et al. [22], the connection between the temperature and asphalt mastics’ viscosity can be accurately described by the Arrhenius equation, as shown in Equation (1):

(1) $$\eta =A\cdot e^{E_{\eta }/RT}$$

(2) $$ln\eta =lnA+\frac{E_{\eta }}{R}\times \frac{1}{T}$$

where *η* denotes viscosity, *A* denotes regression coefficient, *R* is the universal gas constant (R = 8.314 J/mol k), *T* denotes the absolute temperature, and *E* denotes flow activation energy. The larger *E_η_* represents the more energy required for the flow of asphalt mastics, indicating that asphalt mastics are more difficult to flow and less sensitive to temperature changes.

**Figure 8:**
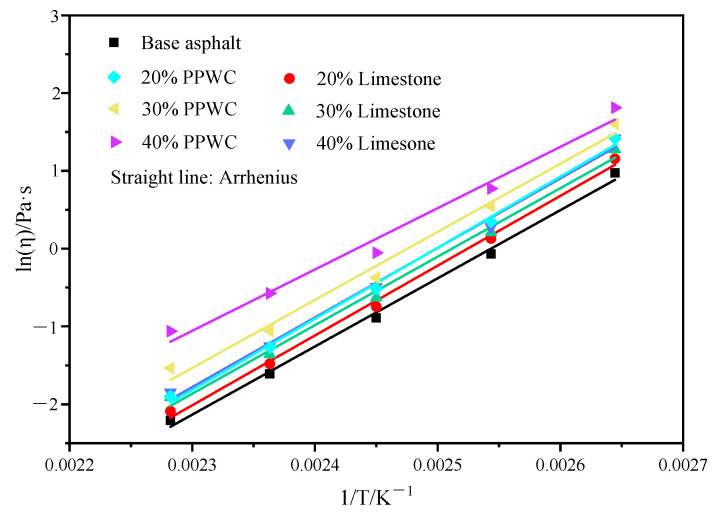
Viscosity–temperature curve of asphalt mastics.

The viscosity–temperature curve can be built using Equation (2) using the viscosity of asphalt mastics at various temperatures, as shown in Figure 8. Based on the viscosity–temperature curve, Table 5 lists the viscosity–temperature equation as well as the flow activation energy of asphalt mastics. All viscosity–temperature equations have linear correlation coefficients (R^2^) greater than 0.98, indicating that Equation (1) can accurately represent the connection between asphalt mastics’ viscosity and temperature. The results suggest that the incorporation of filler will increase the flow activation energy of the asphalt mastics. When the filler content is the same, asphalt mastics with PPWC filler have a lower *E_η_* than asphalt mastics with limestone filler, which suggests that they are more sensitive to temperature, that is, the viscosity changes more rapidly with temperature.

### 4.3. Rheological Properties of Asphalt Mortar

**Figure 9:**
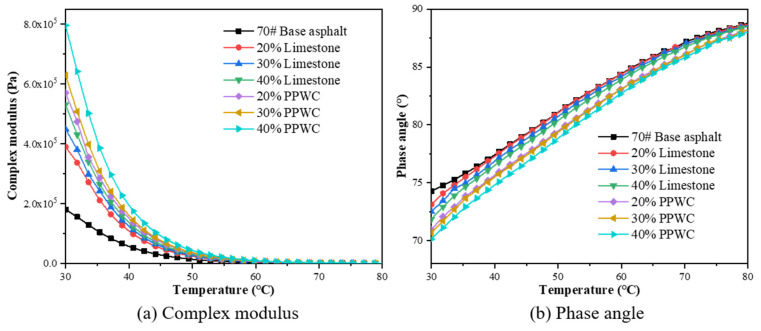
The complex modulus and phase angle of asphalt mastics by temperature sweep test.

The ambient temperature has a considerable impact on the rheological characteristics of asphalt. This study used DSR test to examine the rheological characteristics of asphalt mastics at various temperatures. The complex modulus (G*) and phase angle (δ) of the asphalt mastics at high temperatures were measured using the temperature sweep test, as shown in Figure 9. G* represents the resistance to deformation of asphalt mastics. The higher G* means that the asphalt mastics are less susceptible to deformation under load. δ reflects the viscoelastic behavior of the asphalt mastics. The smaller δ indicates that the more elastic parts in the viscoelasticity of asphalt mastics and the stronger ability to restore deformation.

It can be observed from Figure 9a that as the temperature increases from 30 °C to 80 °C, the G* of the asphalt mastics decreased, indicating that higher temperatures soften the asphalt mastics. However, the G* of the asphalt mastics rose with the increase in filler content at the same temperature, demonstrating that the incorporation of filler had a hardening impact on the base asphalt. When the filler content is the same, compared to the limestone filler asphalt mastics, the G* of the PPWC filler asphalt mastics was much greater, demonstrating that PPWC filler asphalt mastics have the higher deformation resistance than limestone filler asphalt mastics.

The curves in Figure 9b demonstrate that the asphalt mastics’ δ increased as temperature rose, indicating the transition from an elasticity-dominated stage to a viscosity-dominated stage; that is, the proportion of viscous deformation in deformation increases. It is evident that the addition of filler improved the elastic component of asphalt mastics viscoelasticity since, at the same temperature, δ decreased with an increase in filler content. Furthermore, the enhancement of PPWC filler is more significant than limestone filler. This might be explained by the PPWC filler’s lower particle size and rougher surface. Zhao et al. [35]’s study shows that the filler with rough surface can increase the internal friction between filler particles and asphalt, thus increasing the elasticity of asphalt mastics.

**Figure 10:**
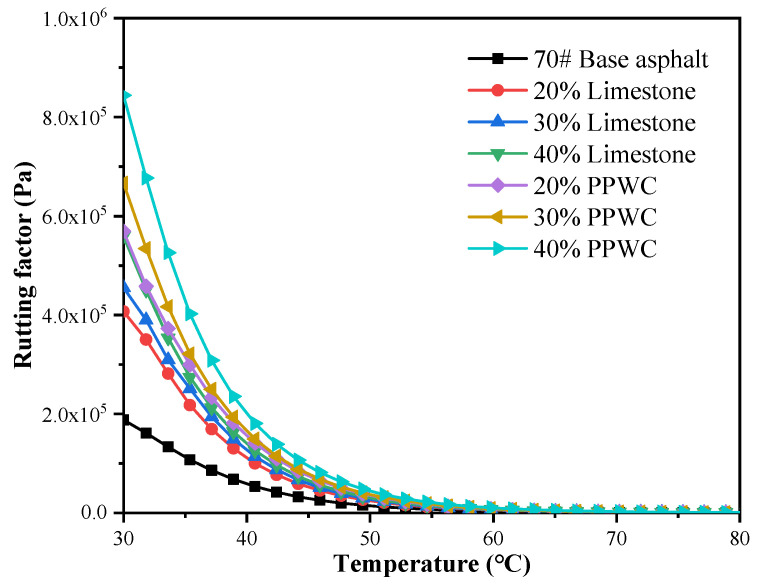
The rutting coefficient of asphalt mastics.

The rutting coefficient can be used to objectively examine the rutting resistance of asphalt mastics, which is defined as the ratio of G* and sinδ. Asphalt mastics exhibit greater rutting resistance as rutting coefficient increases. According to the standard AASHTO [54], the G*/sinδ of base asphalt should be higher than 1 kPa at the highest pavement design temperature. The rutting coefficient of the asphalt mastics generated by the two fillers is depicted in Figure 10. The rutting coefficient obviously dropped as the temperature rose, and the incorporation of filler can strengthen the asphalt mastics’ resistance to rutting. The PPWC filler asphalt mastics had a much greater rutting coefficient than the limestone filler asphalt mastics when the filler content was the same, indicating that using PPWC filler to replace limestone filler can enhance the anti-rutting performance of the asphalt mastics.

**Figure 11:**
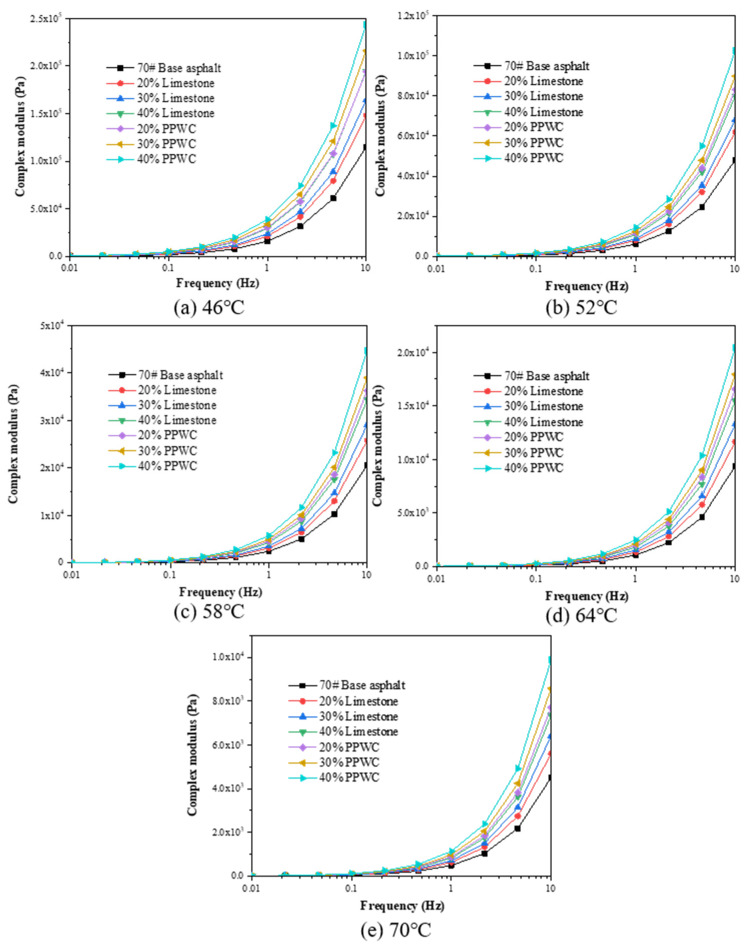
The complex modulus of asphalt mastics by frequency sweep test.

It has been reported that the frequency sweep test can be used to investigate the viscoelastic response of asphalt mastics under dynamic load [39]. The results of complex modulus (G*) obtained by frequency sweep test at five temperatures are shown in Figure 11. It is evident that G* decreased with the increase of temperature. G* rose with increasing loading frequency and filler content at the same temperature, showing that the addition of filler will improve the asphalt mastics’ elasticity under dynamic stress. When the filler content is the same, the G* of PPWC filler asphalt mastics was significantly higher than limestone asphalt mastics, showing that using PPWC filler to replace limestone filler can improve the elastic characteristics of asphalt mastics.

### 4.4. BBR Test Results

**Figure 12:**
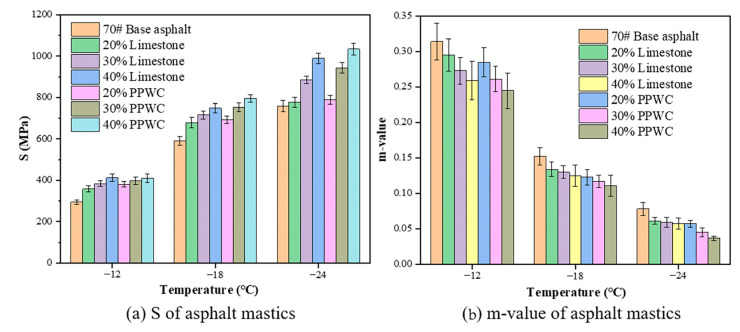
The S and m values of asphalt mastics by BBR test.

To assess the low-temperature crack resistance of asphalt mastics, BBR test was conducted. A smaller S and a larger m value under low-temperature conditions show that the asphalt mastics have better adaptation to deformation and stronger crack resistance. The S and m values of asphalt mastics with various filler contents at −12 °C, −18 °C, and −24 °C are shown in Figure 12. It can be observed that the S of asphalt mastics increased, and the m value fell as the filler content increased, demonstrating that the filler has a detrimental effect on base asphalt’s resilience to low-temperature cracking. The S of PPWC filler asphalt mastics was higher than that of limestone filler asphalt mastics when the filler-to-asphalt volume ratio was the same, and the m value was lower than that of limestone filler asphalt mastics. As a result, it has inferior low-temperature crack resistance than asphalt mastics with limestone filler. This might be as a result of the PPWC filler’s irregular shape and rough surface. Rougher surface can increase the internal friction between the filler and asphalt [35]. As a result, the interlocking effect in asphalt mastics between asphalt and PPWC filler is stronger than that between asphalt and limestone filler, increasing the stiffness of asphalt mastics and degrading low-temperature performance.

### 4.5. Economic Analysis of PPWC Filler

Based on the following factors, grinding PPWC into particles smaller than 0.075 μm to replace limestone filler can reduce the production cost of asphalt concrete:

(1) By grinding treatment, PPWC can be reused, reducing waste emissions and complying with environmental requirements. This not only reduces the cost of waste disposal but also maximizes resource utilization, thereby lowering overall costs.
(2) PPWC, as a solid waste, does not require a mining process, whereas limestone extraction adds additional costs. Therefore, the processing and utilization costs of PPWC are lower, thus reducing the raw material costs.
(3) PPWC is typically generated near construction sites. Therefore, using PPWC filler can reduce the transportation distance of raw materials, leading to decreased transportation costs.
(4) In some regions, the government has policies and regulations related to waste management and resource utilization. Grinding PPWC for reuse complies with these policies and regulations. This approach might garner government support and incentives, ultimately lowering production costs.

## 5. Conclusions

This study aims to evaluate the feasibility of using PPWC filler to replace limestone filler in asphalt mixture. Firstly, limestone and PPWC fillers’ chemical and physical characteristics were examined. Then, asphalt mastics with various filler-to-asphalt volume ratios were prepared using two different types of fillers, and their physical and rheological characteristics were examined. Based on the test results, the main conclusions are as follows:

(1) Compared to limestone filler, PPWC filler has a lower density (2.67 g/cm^3^), smaller particle size (12.54 μm) and rougher surface, and its hydrophilicity coefficient (1.77) is higher than limestone filler (1.44).
(2) As the filler-to-asphalt volume ratio increased from 0% to 40%, the softening point of the asphalt mastics containing PPWC filler improved by 15.1%, but the penetration and ductility decreased by 38.9% and 47.4%, respectively.
(3) In comparison to the asphalt mastics with limestone filler, the performance of the asphalt mastics’ rutting resistance and high-temperature stability can both be greatly improved by PPWC filler. However, the performance of PPWC filler asphalt mastics at low temperatures is less impressive.
(4) Considering that PPWC filler will reduce the low-temperature performance of asphalt mastics, it is suggested that the content of PPWC filler should not exceed 30% of asphalt volume.

The above conclusions show that PPWC filler can be used to replace limestone filler in asphalt mixtures. Replacing limestone filler with PPWC filler can maximize resource utilization, thereby providing a new approach to the development of eco-friendly road infrastructure. In the ongoing study, PPWC filler will be used to replace limestone filler to prepare asphalt concrete and study its road performance.

## Figures and Tables

**Table 1:** Basic properties of asphalt.

Properties	Test Value	Standard Requirement JTG E20-2011
Density/(g/cm^3^)	1.034	
Penetration (25 °C)/0.1 mm	69.2	60–80
Softening point/°C	46.5	≥46
Ductility 5 cm/min, (15 °C)/cm	186	≥100
Dynamic viscosity (60 °C)/(Pa·s)	242.4	≥100

**Table 2:** Basic properties of limestone filler and PPWC filler.

Properties	Density/(g/cm^3^)	Hydrophilic Coefficient
Limestone	2.71	0.74
PPWC	2.67	0.79
Standard requirement	≥2.50	≤1.00

**Table 3:** Particle size distribution of PPWC filler and limestone filler.

Filler	d (0.1) μm	d (0.5) μm	d (0.9) μm	Specific Surface Area (m^2^/g)
PPWC	1.31	7.58	34.67	1.77
Limestone	1.66	8.71	39.81	1.44

**Table 4:** Oxide composition of PPWC filler and Limestone filler.

Oxide Type	Filler	CaO	SiO_2_	MgO	Al_2_O_3_	Na_2_O	SO_3_	CO_2_
Oxide content (%)	PPWC	35.10	32.40	1.11	5.84	0.38	3.69	15.92
	Limestone	56.92	18.35	1.73	7.62	0.13	2.12	8.82

**Table 5:** The viscosity–temperature equation and flow activation energy of asphalt mastics.

Samples	Fitting Line	R^2^	Slope (E_η_/R)	E_η_ (kJ/mol)
Base asphalt	y = 8763.1x − 22.3	0.9960	8763.1	72.78
20% PPWC	y = 8939.1x − 22.8	0.9966	8939.1	74.32
20% Limestone	y = 8973.7x − 22.7	0.9967	8973.7	74.61
30% PPWC	y = 8754.1x − 21.7	0.9890	8754.1	72.86
30% Limestone	y = 8807.8x − 22.1	0.9938	8807.8	73.23
40% PPWC	y = 8796.2x − 19.2	0.9840	8796.2	73.13
40% Limestone	y = 8940.8x − 22.3	0.9938	8940.8	74.33

## Data Availability

The data presented in this study are available on request from the corresponding author.
